# Object Detection Based on the GrabCut Method for Automatic Mask Generation

**DOI:** 10.3390/mi13122095

**Published:** 2022-11-28

**Authors:** Hao Wu, Yulong Liu, Xiangrong Xu, Yukun Gao

**Affiliations:** School of Mechanical Engineering, Anhui University of Technology, Maanshan 243032, China

**Keywords:** deep learning, object detection, image segmentation, Mask R-CNN

## Abstract

The Mask R-CNN-based object detection method is typically very time-consuming and laborious since it involves obtaining the required target object masks during training. Therefore, in order to automatically generate the image mask, we propose a GrabCut-based automated mask generation method for object detection. The proposed method consists of two stages. The first stage is based on GrabCut’s interactive image segmentation method to generate the mask. The second stage is based on the object detection network of Mask R-CNN, which uses the mask from the previous stage together with the original input image and the associated label information for training. The Mask R-CNN model then automatically detects the relevant objects during testing. During experimentation with three objects from the Berkeley Instance Recognition Dataset, this method achieved a mean of average precision (mAP) value of over 95% for segmentation. The proposed method is simple and highly efficient in obtaining the mask of a segmented target object.

## 1. Introduction

In the field of computer vision, object detection is a basic technique that combines classification and recognition. In recent years, it has been applied in several ways, including automatic driving, robotic grabbing, and face recognition. Various factors can disrupt the detection process, such as incorrect angles, occlusion, and uneven light. Traditional object recognition methods involve manually designing some features, such as the histogram of oriented gradients (HOG) feature [1], the scale-invariant feature transform (SIFT) [2], and the deformable part-based model (DPM) [3].

In recent years, emerging deep learning techniques have also been applied in the field of object recognition. First, Krizhevsky [4] proposed a large-scale deep neural network called AlexNet and implemented the classification technology in the ImageNet dataset, following which many new types of deep neural networks were proposed for object recognition. The deep neural network for object detection can be divided into one-stage detection and two-stage detection depending on the structure [5]. The former directly generates and finds objects in the network after inputting the image. Examples of such algorithms include YOLO [6] and SSD [7]. By contrast, the latter approach involves extracting the features of the convolutional neural network (CNN) after inputting the image and then predicting the classification and position of the object. Representative algorithms include the R-CNN series [8,9,10].

The YOLO algorithm, proposed by Redmon et al. [6], is a CNN that can predict multiple box positions and categories simultaneously. The network design approach of the YOLO algorithm extends the core idea of GoogleNet Although it can perform end-to-end target detection and is less time consuming, its accuracy has declined. 

The SSD algorithm, proposed by Liu et al. [7], is a single-layer deep neural network that can be applied for multi-class object detection. It involves using a small convolution filter to predict a set of default bounding box category scores and box offsets in the feature map.

Girshick et al. [8] proposed the R-CNN model, which uses Selective Search to obtain candidate regions (approximately 2000 regions). The size of the candidate area is then normalized and used as the standard input to the CNN network. Then, AlexNet is used to identify the features in the candidate area; finally, multiple support vector machines (SVMs) are used to classify and fine-tune the positioning box.

In 2016, Ren et al. [11] proposed the Faster-R-CNN algorithm, which introduces RPN to extract proposals. RPN is a fully convolutional neural network and shares the features of the convolutional layer. Therefore, it can realize the extraction of a proposal. The core idea of RPN is to use the CNN to generate region proposals directly by using a sliding window. RPN only needs to slide on the last convolutional layer because the anchor mechanism and box regression can be used to obtain region proposals with multi-scale aspect ratios.

**Figure 1:**
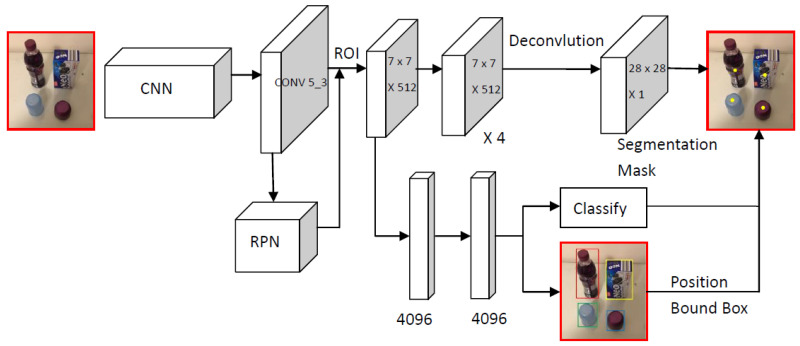
Ref. [15]. The Mask R-CNN network.

Mask R-CNN [12] is an improvement on Faster R-CNN because it focuses on instance segmentation. In addition to classification and positioning regression, this algorithm adds parallel branches for instance segmentation and jointly trains their losses. The detailed structure of the algorithm is shown in Figure 1. The Mask R-CNN network has two main parts, of which the first is RPN. After the alignment using ROIAlign, the second part begins, which includes the segmentation mask prediction network. The main structure of the network uses VGG [13]. RPN connects to the last convolutional layer of VGG and produces the RoI as the output. Then, the feature extraction is performed and pooled to a fixed size. These pooled features are used as branch inputs. For the network’s positioning and classification branches, an architecture consisting of fully connected layers, convolution layers, and deconvolution layers is used. For the segmentation branch, the target object is accurately segmented through an architecture composed of multiple convolutional layers, deconvolution, and a segmentation mask. Therefore, the object detection method based on Mask R-CNN has three different tasks branches, namely, positioning, classification, and object segmentation, which aim to achieve the classification, positioning, and segmentation of objects simultaneously.

Mask R-CNN has achieved very satisfactory results in the classification of object instances. However, it is very laborious to obtain the required object masks for training, such as in using the LabelMe annotation tool (http://labelme.csail.mit.edu/Release3.0/, accessed on 15 September 2022). Therefore, we propose a new method based on the GrabCut method by which to automatically mark and obtain image masks to train deep learning models. The proposed method consists of two stages: The first stage is based on GrabCut’s interactive image segmentation method by which to generate masks [14]. The second stage involves using the GrabCut output of the mask for detection.

This paper is organized as follows: Section two briefly describes the automatically generating image mask method, followed by the experimental results as well as the discussion and conclusions. 

## 2. Automated Generating Image Mask Method 

**Figure 2:**
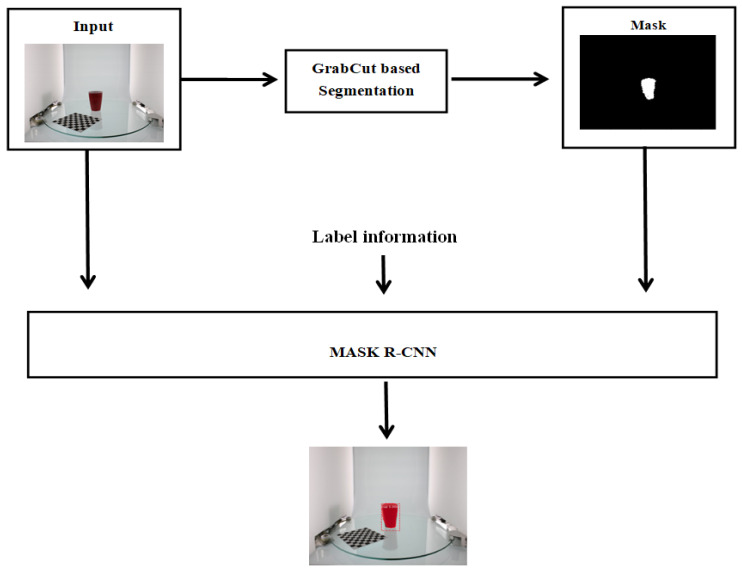
Automated method for generating the image mask.

As shown in Figure 2, the proposed method consists of two parts. The first part implements GrabCut-based interactive image segmentation. This process yields a pixel-level segmentation result, which is the mask of the image. In the second part, Mask R-CNN-based object detection is performed, in which the image mask, the original input image, and the image label information, such as the object type and background, are used for training. The outputs include the object segmentation results, label information, and average value of precision.

### 2.1. GrabCut-Based Mask Segmentation

In this paper, using GrabCut to perform the segmentation task, we must first manually frame and select the target area, which automatically segments the possible target area, and then conduct a small amount of user interaction, that is, specify that some pixels belong to the target, and that the cut image will be a color image with the background removed. The removed target area image needs to be converted into a black-and-white–gray image, which is more convenient for image processing as an image mask.

GrabCut is an improvement on the iterative Graph Cut algorithm [16] and is an iterative minimization algorithm. Each iteration in the process decides each parameter of the Gaussian mixture model (GMM) to make the segmentation between the object and the background where it is easier to perform so that the image segmentation also makes the final segmentation result look better from the effect.

According to Rother [14], the GrabCut algorithm uses texture (color) information and boundary (contrast) information in the image. Therefore, this algorithm requires only a small amount of user interaction or simple frame selection and labeling to obtain better segmentation results.

The GrabCut algorithm first requires the user to simply select the foreground and background to establish a GMM on the foreground and background area. Then, it initializes the GMM using the *k*-means algorithm to calculate the distance from the nodes to the foreground or background and the distance between adjacent nodes. Based on this information, it obtains the split energy weight, constructs the s-t network graph for the unknown area, and uses the maximum flow/minimum cut algorithm to split it. The segmentation process of the GrabCut algorithm involves continuously updating and modifying the GMM parameters through iterations so that the algorithm tends to converge. Because the group parameters of the GMM are optimized during the iteration process, the segmentation energy is gradually reduced. Finally, it is ensured that the segmentation energy converges to the minimum value and image segmentation is realized.

**Figure 3:**
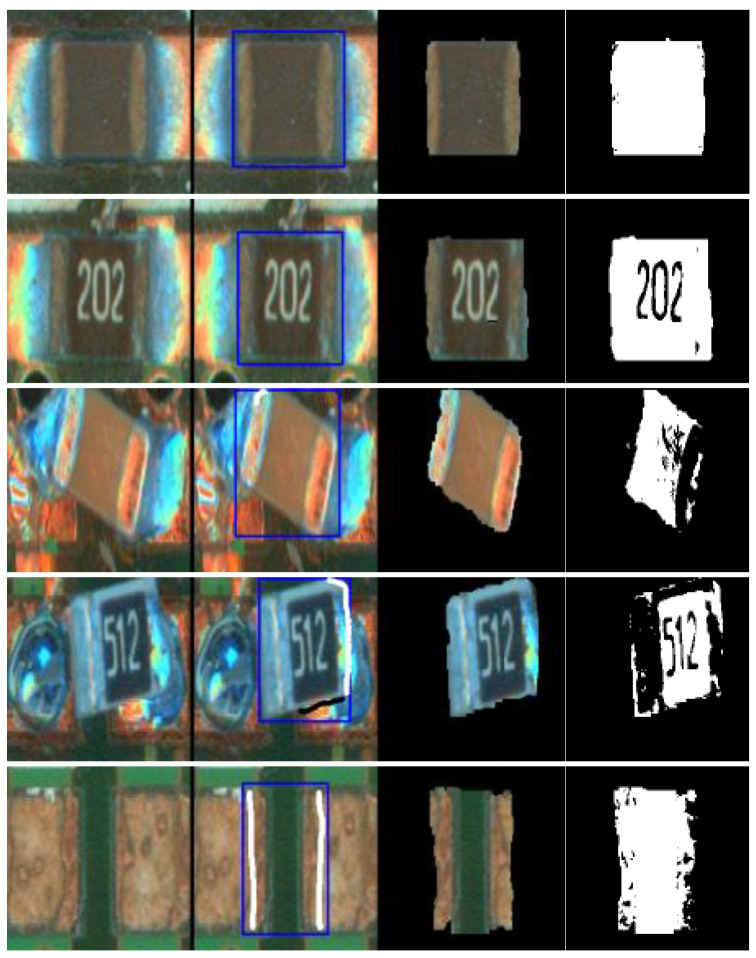
Operation for GrabCut base component segmentation methods.

The specific process is as follows: When running the GrabCut algorithm with PyCharm, an interactive interface pop ups. The instructions are then followed to process the image in the interactive interface. First, the target area must be manually box selected in the image. The algorithm automatically segments the possible target area according to the box selected area. If the segmentation effect is poor and the target area is not segmented or the background is wrongly segmented into the target area, we can enter the subsequent interactive operation, mark the target area or background with a simple line, and then execute the segmentation algorithm to achieve the goal of the semi-automatic segmentation of the target area. According to the minimum energy method, the algorithm can segment the pixels that approximate the target area to achieve interactive GrabCut. Due to the increase in manual intervention, it is more accurate than automatic segmentation. The following is a representative image that marks five types of electronic components and provides the operations required for segmentation as shown in Figure 3. Here, the required mask can be obtained by simply framing and labeling the target area. The final mask result is shown in Figure 3.

### 2.2. Object Detection Based on Mask R-CNN Method

The proposed object detection method based on Mask R-CNN has three branches that perform different tasks, namely, the bounding box positioning branch, bounding box classification branch, and segmentation branch. The positioning and classification branches of the bounding box directly use the fully connected layer to obtain the results. The segmentation branch mainly includes continuous convolution, deconvolution, and a segmentation mask. More specifically, it first obtains the Feature Maps from the labeled training dataset through the FPN network, following which they are fed into RPN to obtain the region proposals. These are input into the ROIAlign module to extract the region of interest, which is then inputted to two branches of the segmentation branch and the regression classification network. While the former receives the target mask and segmentation results, the latter receives the results of the classification and positioning of the box area. In order to train our proposed detection method, we use a platform consisting of Python and TensorFlow-GPU. The network is first initialized and trained on the Microsoft COCO dataset [17] after pre-training on the same dataset, and the detection method is then fine-tuned on our dataset.

## 3. Implementation Details

### 3.1. Selection of Dataset

**Figure 4:**
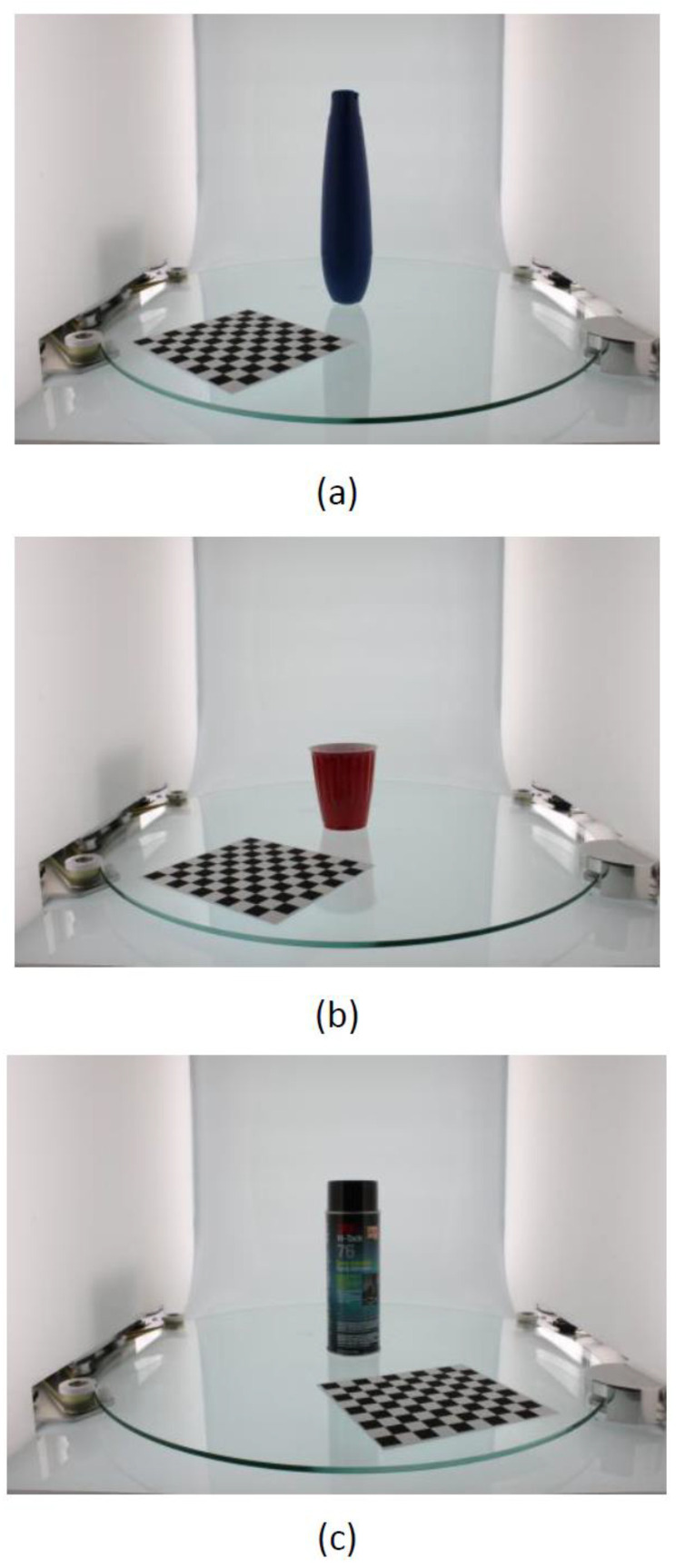
Dataset for experiment [18]: (**a**) ikea_table_leg_blue; (**b**) ikea_table_red_cup; (**c**) 3 m_high_tack_spray_adhesive.

In this experimental design, the choice of dataset is very important. The dataset used in this experiment was obtained from BigBIRD: Big Berkeley Instance Recognition Dataset [18]. Three datasets were arbitrarily selected from a series of original experimental datasets, which are shown in Figure 4, namely, the ikea_table_leg_blue, ikea_table_red_cup, and 3 m_high_tack_spray_adhesive datasets. Each dataset was divided into five types of pictures, which were the datasets obtained from five directions by the sensor.

### 3.2. GrabCut Algorithm to Obtain the Mask

The size of the dataset we obtained was not convenient for experiments. In order to improve the efficiency of the GrabCut algorithm when segmenting images, we needed to adjust the sizes of the pictures. We chose to resize the images using Python3. The resolution of the images was adjusted from 4272 × 2848 pixels to 224 × 224 pixels. After resizing, we used the GrabCut algorithm to segment the images and obtain the object masks.

**Figure 5:**
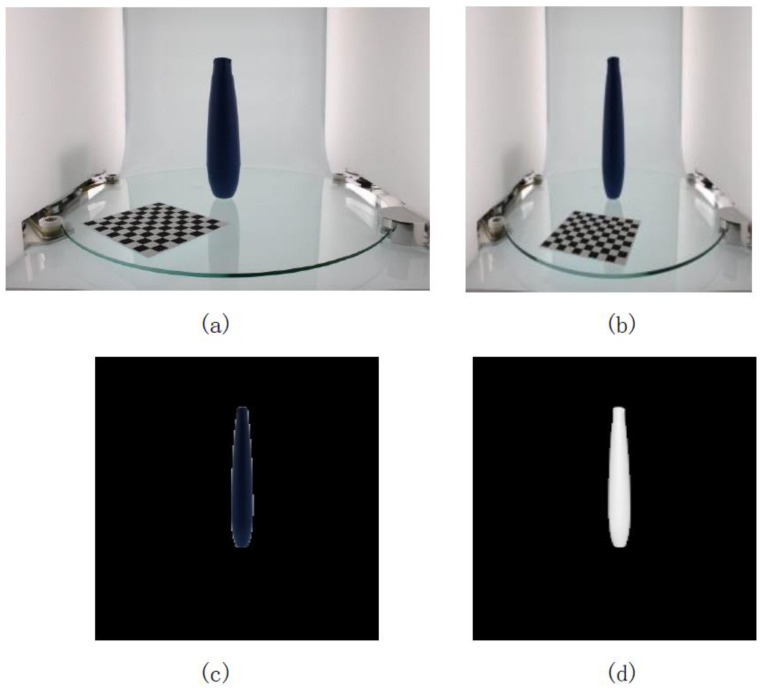
(**a**) Original image with 4272 × 2848 pixel; (**b**) image after resizing to 224 × 224 pixel; (**c**) segmentation results based on GrabCut; (**d**) binarization results of the mask.

The experimental steps of using the GrabCut algorithm on the images is described below. First, one or more rectangles containing objects in the image were defined and the area outside the rectangles was automatically regarded as the background. For a user-defined rectangular area, the data in the background could be used to differentiate between the foreground and background areas. The GrabCut algorithm used a GMM to model the background and foreground and mark undefined pixels as potential foreground or background. The schematic diagram of the entire process is shown in Figure 5. Figure 5a shows original image data with a resolution of 4272 × 2848 pixels and Figure 5b shows the resized image with a resolution of 224 × 224 pixels. Figure 5c shows the GrabCut algorithm used for interactive image segmentation to obtain the segmented object mask. Figure 5d is the corresponding binary image that was obtained after the GrabCut-based segmentation.

### 3.3. Mask R-CNN-Based Object Detection

Before using the Mask R-CNN algorithm for object detection, it is important to prepare the dataset for training. Three files are required for this purpose, including the original input image, the image masks, and the annotation file. GrabCut has previously been used to obtain object masks, as shown in Figure 5c, based on the original input image, as shown in Figure 5b. Annotation data can be prepared by writing object and background information.

**Figure 6:**
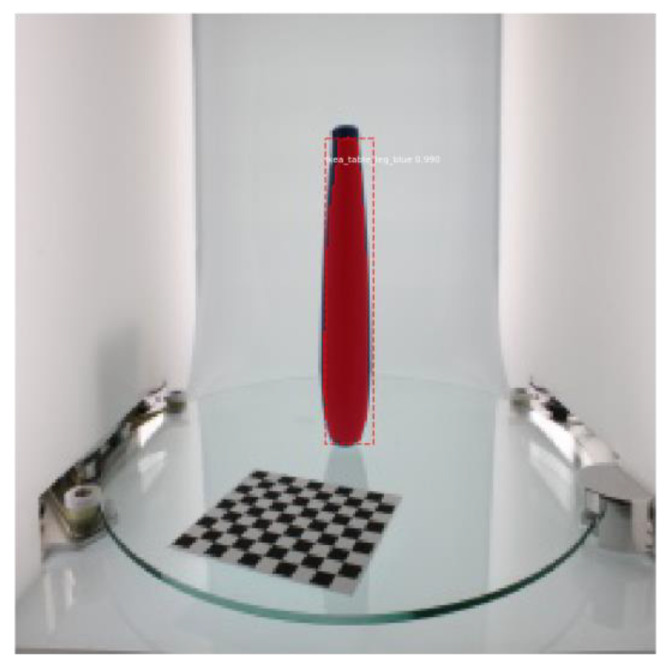
Object detection results using the Mask R-CNN method.

After the input data was prepared, the data needed to be trained. The trained model was saved along with the dataset for testing. Figure 6 shows the results of object detection based on the Mask R-CNN algorithm.

## 4. Results

### 4.1. GrabCut-Based Mask Segmentation

**Figure 7:**
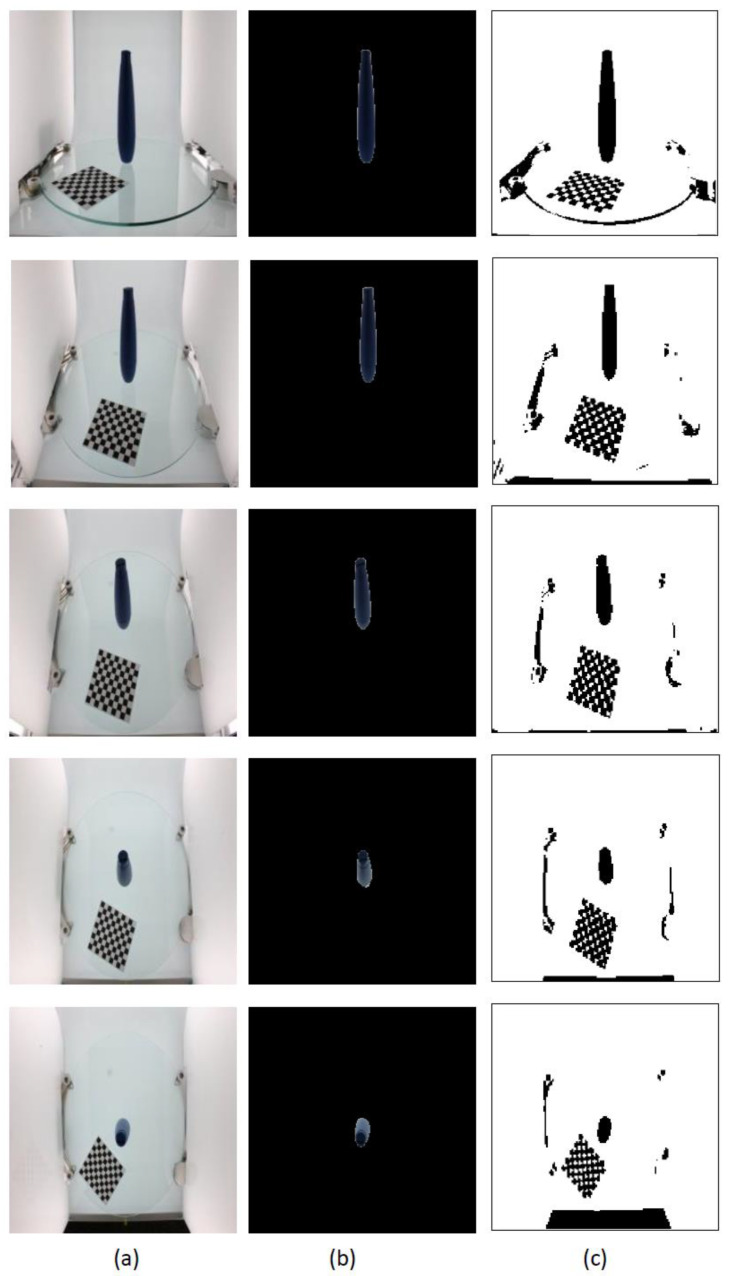
GrabCut-based segmentation experiment results for ikea_table_leg_blue object: (**a**) input image after resizing; (**b**) GrabCut method segmentation results; (**c**) Otsu method segmentation results.

For the ikea_table_leg_blue image data chosen in this study, the segmentation result of the GrabCut algorithm was analyzed and compared with the segmentation result of the Otsu optimal threshold segmentation algorithm [19]. Figure 7a shows images of the object from five different perspectives. In Figure 7b, the target extracted by the GrabCut algorithm is accurate and the edges are more complete and smooth. In Figure 7c, although the Otsu algorithm can roughly segment the shape of the target bottle, the edges are rough. Since the positioned chessboard and dark stationary elements of the image acquisition platform are extracted as the foreground, the target object segmentation result cannot be obtained in one step.

**Figure 8:**
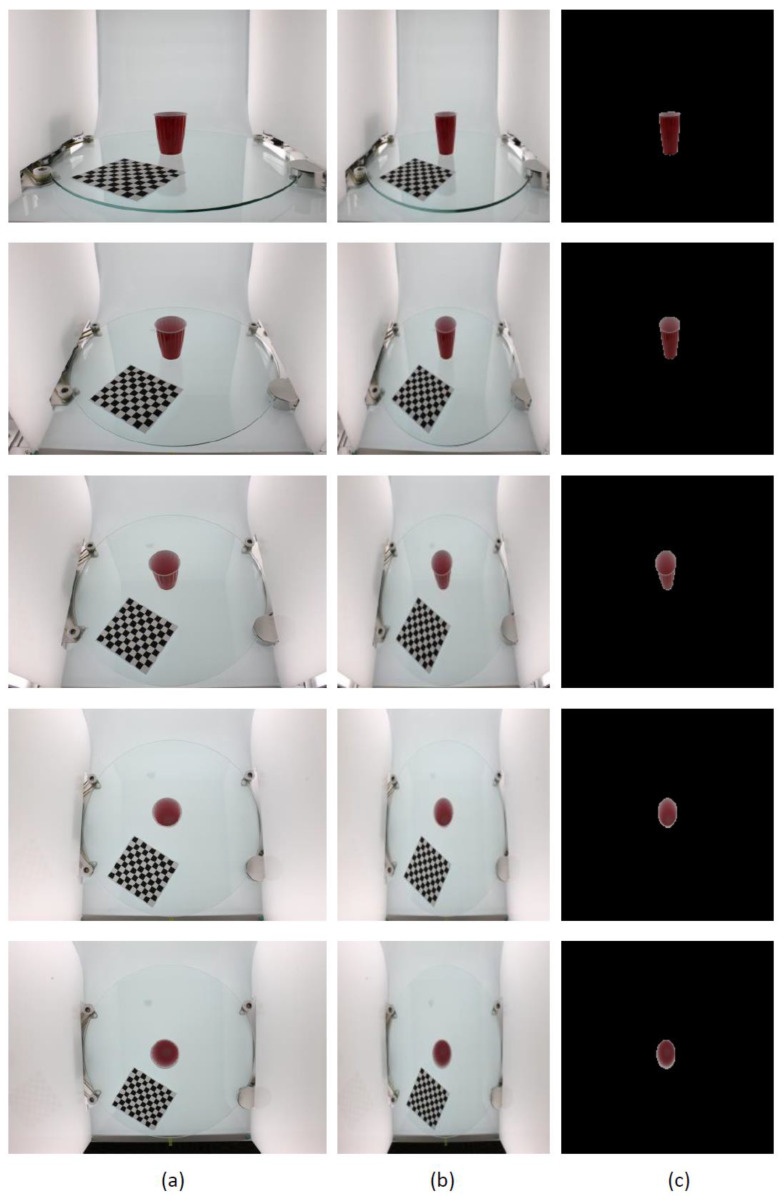
GrabCut-based segmentation experiment results for ikea_table_red_cup object: (**a**) original input image; (**b**) image after resizing; (**c**) GrabCut method segmentation results.

**Figure 9:**
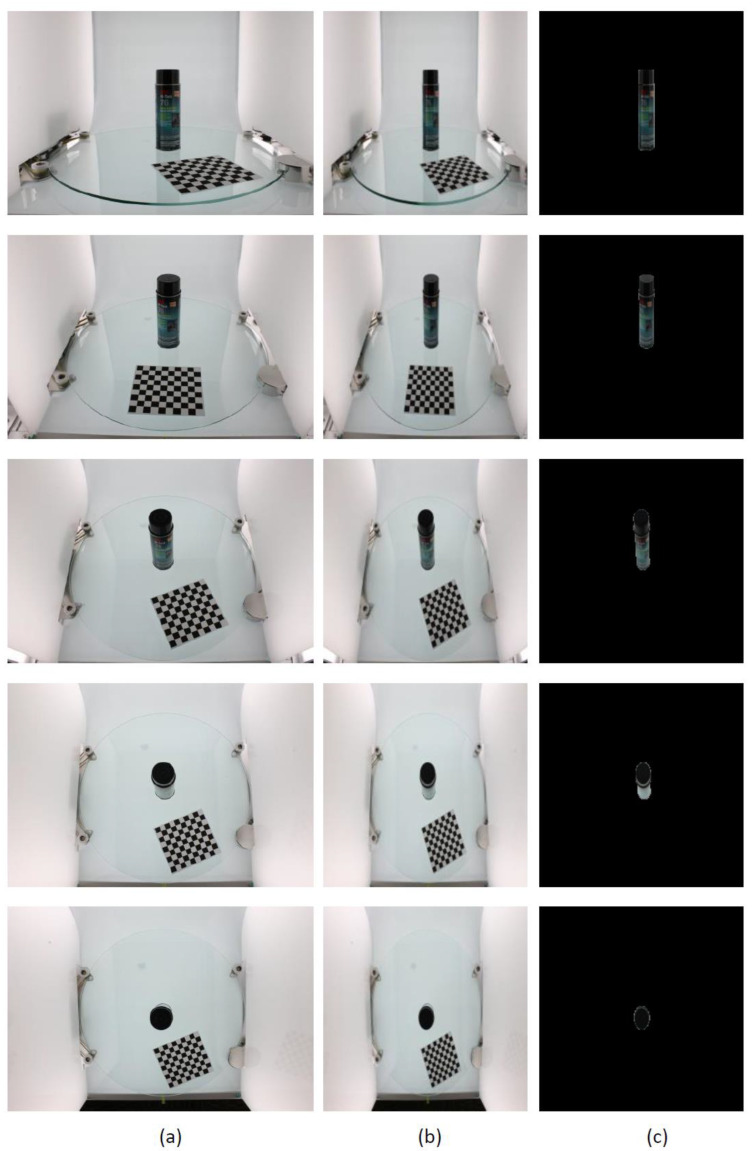
GrabCut-based segmentation experiment results for 3 m_high_tack_spray_adhesive object: (**a**) original input image; (**b**) image after resizing; (**c**) GrabCut method segmentation results.

**Figure 10:**
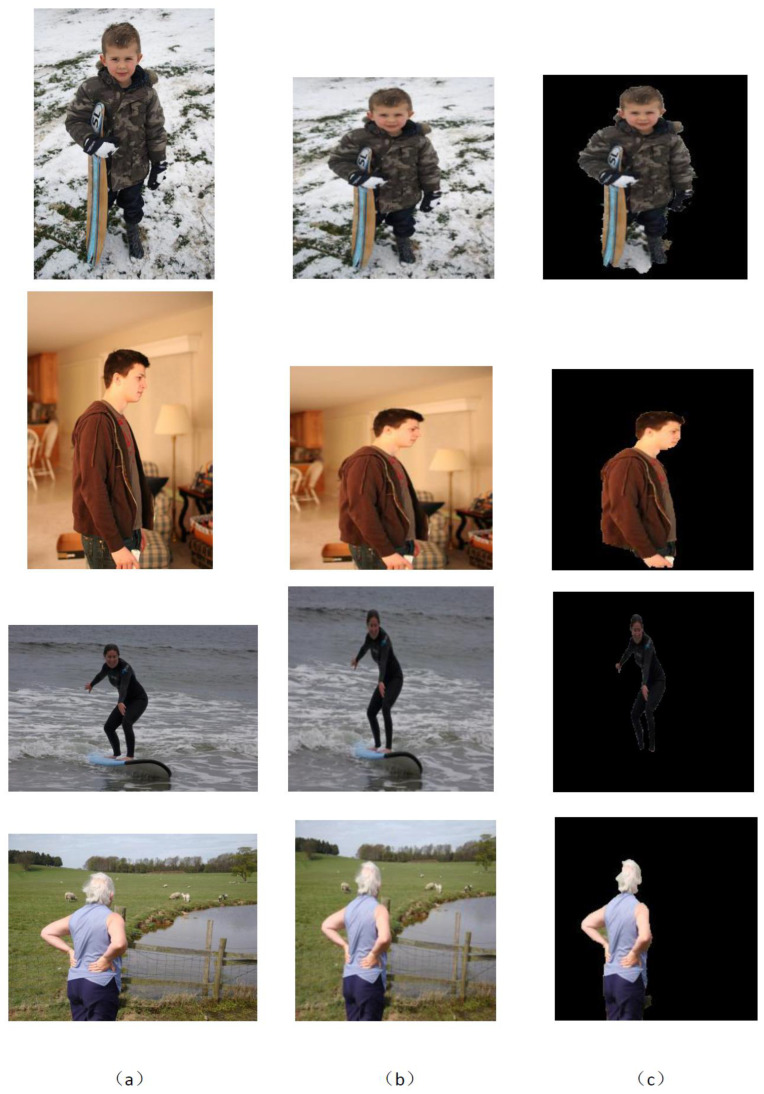
GrabCut-based segmentation experiment results for person objects in COCO dataset: (**a**) original input image; (**b**) image after resizing; (**c**) GrabCut method segmentation results.

**Figure 11:**
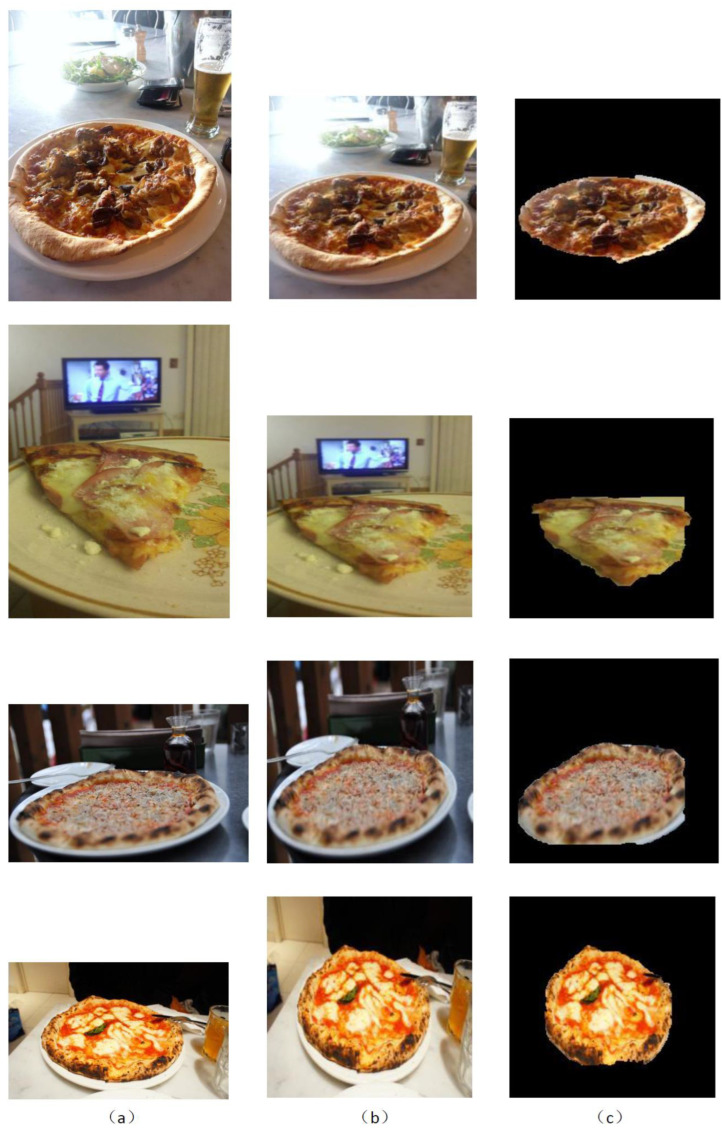
GrabCut-based segmentation experiment results for pizza objects in COCO dataset: (**a**) original input image; (**b**) image after resizing; (**c**) GrabCut method segmentation results.

Further GrabCut segmentation results are shown in Figure 8 for the ikea_table_red_cup data and in Figure 9 for the 3 m_high_tack_spray_adhesive data. The experimental results show that our proposed method can accurately segment the target in the image. We also used our method to segment the COCO dataset, and the results are shown in Figure 10 and Figure 11.

### 4.2. Mask R-CNN-Based Object Detection

The proposed model was evaluated using the software environment and the GPU platform to be configured: (1) Python3.6; (2) Keras 2.0.8; and (3) TensorFlow-GPU. The training process took approximately 15 min, with 30 epochs. The dataset included ikea_table_leg_blue, ikea_table_red_cup, and 3 m_high_tack_spray_adhesive, as shown in Figure 4. Each object had 50 training samples and 5 testing samples.

**Figure 12:**
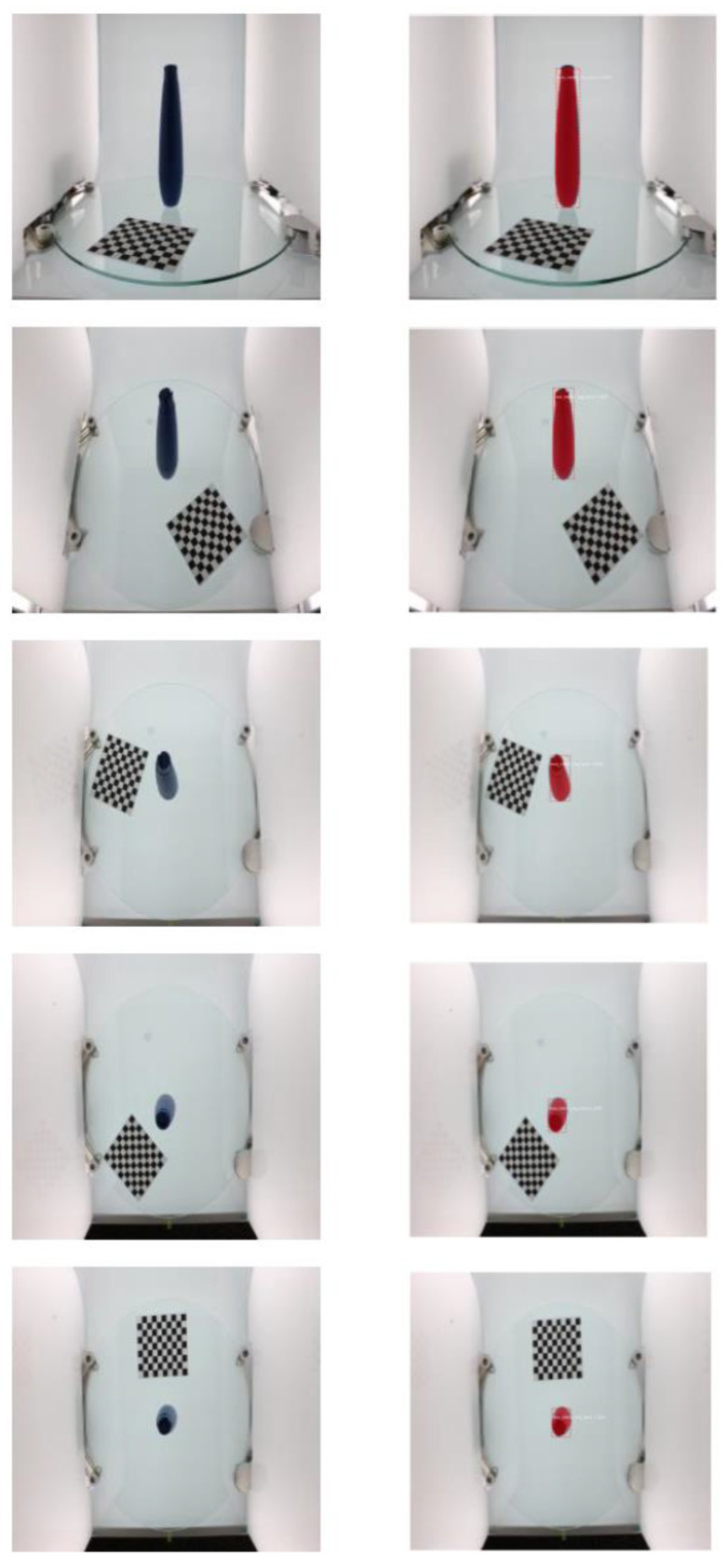
Results for ikea_table_leg_blue data using the Mask R-CNN-based object detection method. The column on the left contains the input images. The column on the right shows the positioning and segmentation results.

**Figure 13:**
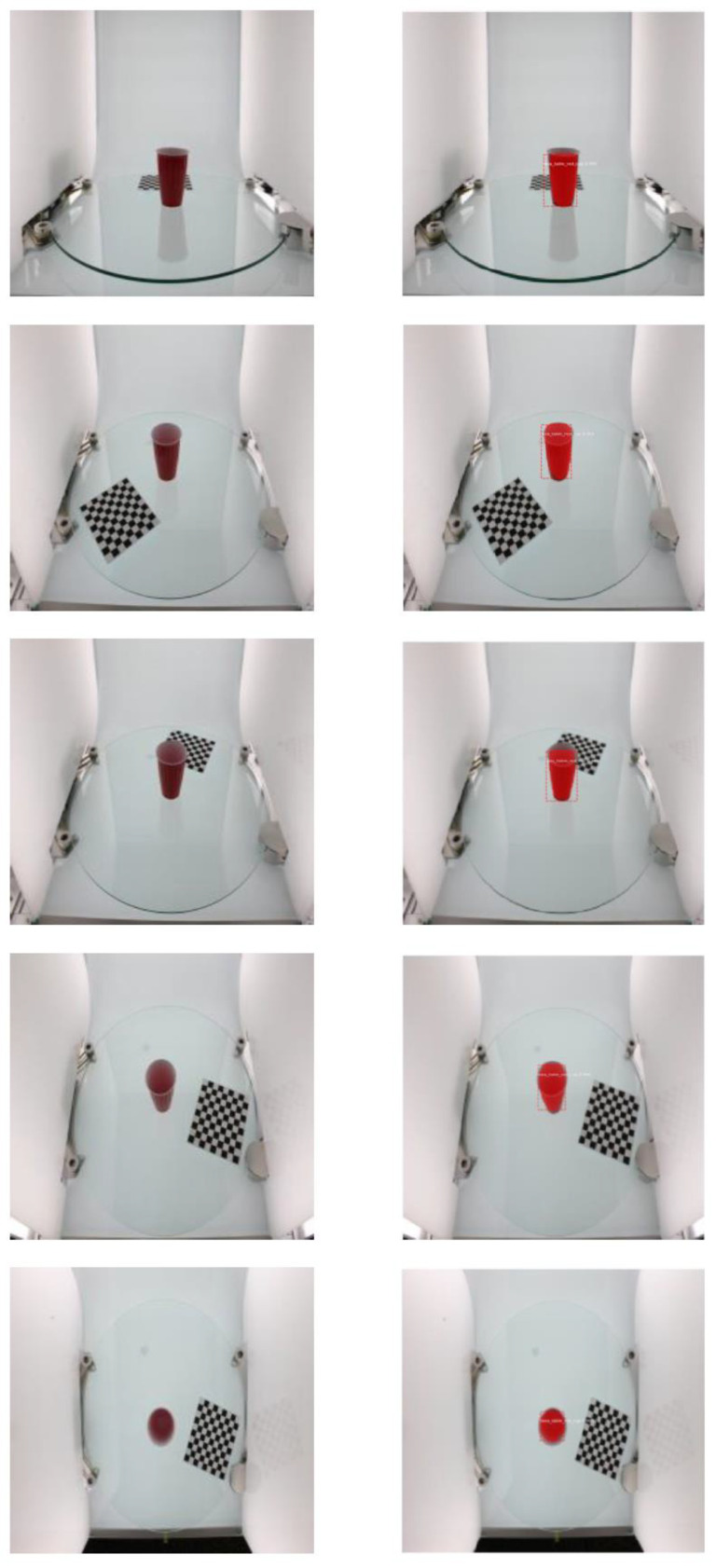
Results for ikea_table_red_cup data using the Mask R-CNN-based object detection method. The column on the left contains the input images. The column on the right shows the positioning and segmentation results.

**Figure 14:**
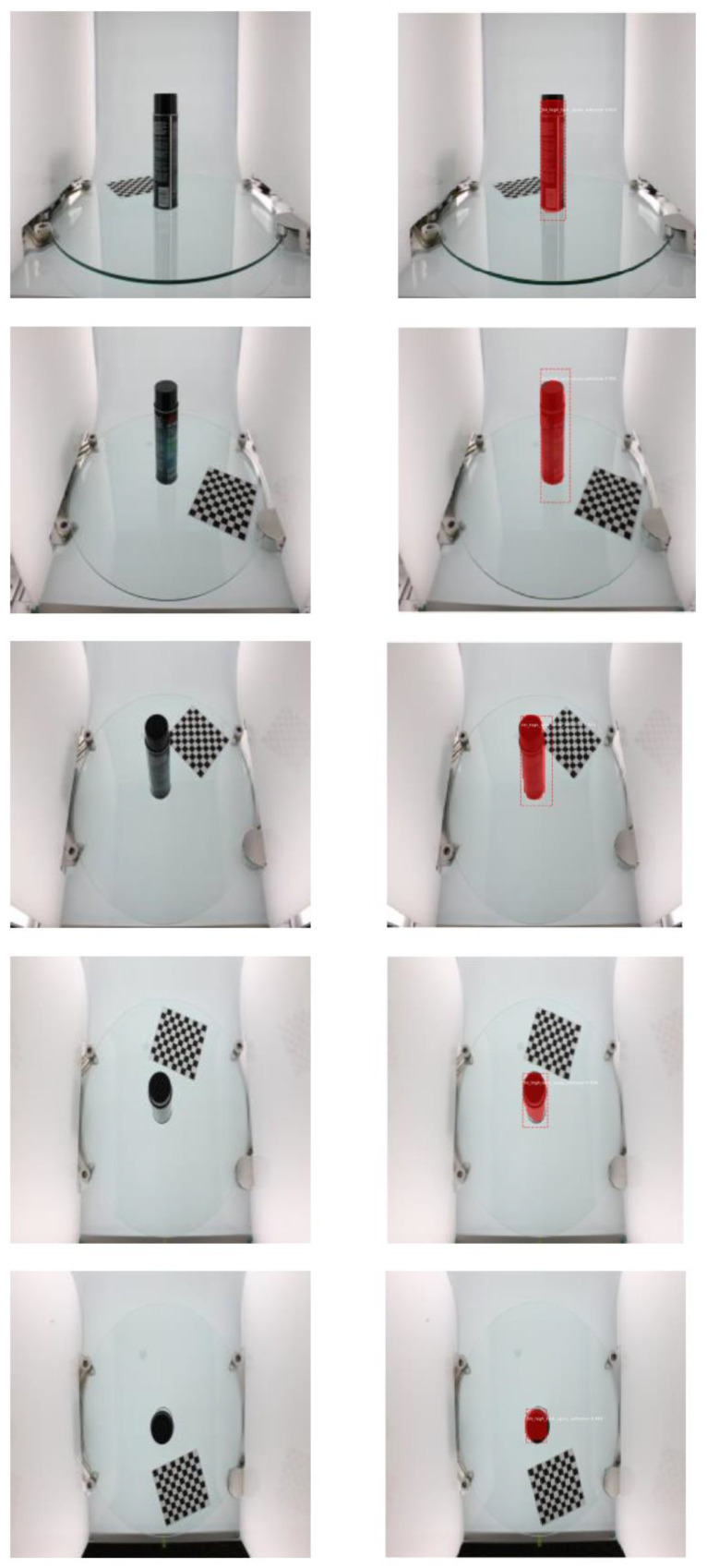
Results for 3 m_high_tack_spray_adhesive data using the Mask R-CNN-based object detection method. The column on the left contains the input images. The column on the right shows the positioning and segmentation results.

**Figure 15:**
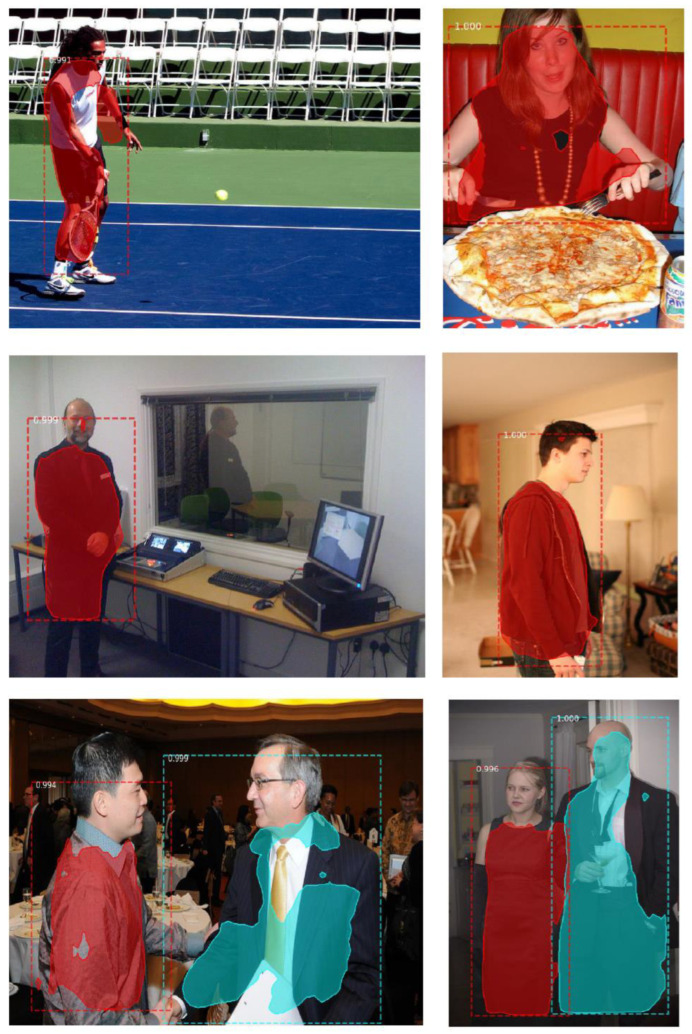
Results for COCO dataset using the Mask R-CNN-based object detection method.

The experimental results of the Mask R-CNN-based object detection method on these three datasets are shown in Figure 12, Figure 13 and Figure 14. In order to further verify the effectiveness of our algorithm, we applied our proposed method to the COCO dataset of target objects with complex and diverse backgrounds and obtained good results, which are shown in Figure 15.

These results show that the methods yielded good segmentation results on these datasets because all the test samples were correctly positioned and segmented. The segmentation accuracy is shown in Table 1. For ikea_table_leg_blue, the mAP_bbox_ value approaches 0.9999 (the average of the same type), the mAP_bbox_ for ikea_table_red_cup is 0.9826, for 3 m_high_tack_spray_adhesive is 0.9816, the mAP_bbox_ for Person object in COCO dateset is 0.998, and the mAP_bbox_ for pizza objects in the COCO dateset is 0.993.

### 4.3. Special Cases: Overlapping Objects and Background

As discussed previously, the proposed GrabCut method can segment an image accurately and obtain the required masks. However, if the target area is simply selected using a frame, some images with interfering pixels cannot be well segmented because the computer is not able to differentiate between similar pixels. Therefore, in the case of more complex images, which contain objects overlapping with the background, other interactive operations need to be added for the user to ensure that the segmentation results include only the object. Examples of such operations include marking some pixel targets as the foreground or background.

**Figure 16:**
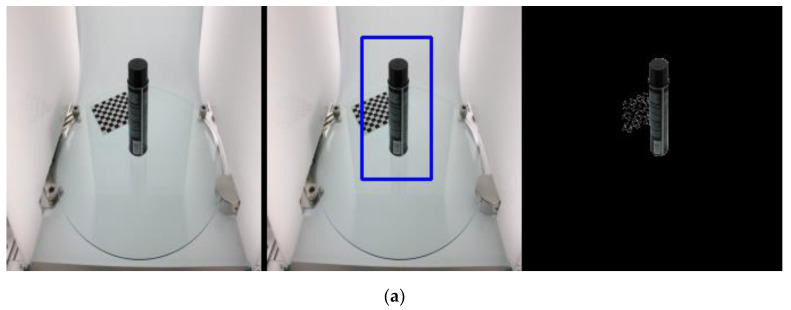

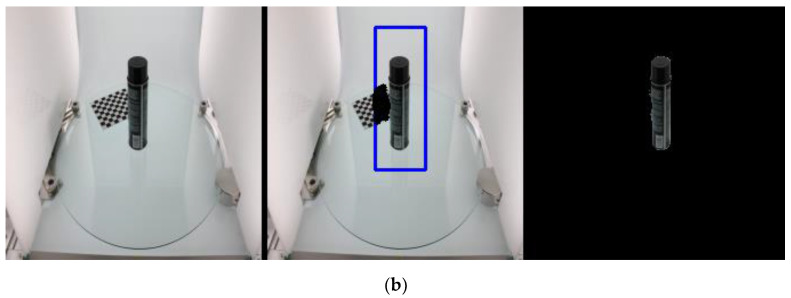
(**a**) Segmentation results with directly selected target area; (**b**) segmentation results with additional marking operation.

As shown in Figure 16a, since the body of the bottle in the 3 m_high_tack_spray_adhesive data has areas with white text, the black area of the body overlaps with the chessboard grid in the background. Therefore, in order to segment the target accurately, an additional step to mark and segment the background or foreground areas is also performed after frame selection. As shown in Figure 16b, the area including the chessboard is blackened and excluded from the target object area.

### 4.4. Comparison of Different Methods of Detection

In order to quantitatively evaluate the performance of the proposed classification method, a comparative experiment to detect electronic components was conducted and compared with traditional classification methods, including SVM, PCA, random forest ensemble learning, and the Mask R-CNN method. Table 2 shows the experimental results. These results show that the proposed method not only achieves the highest accuracy of the Mask R-CNN method, but the mAP index is as high as 98.5%. This could be because the annotated images used in the literature are artificially or forcibly annotated results, which are obtained using LabelMe manual labeling software. By contrast, the method proposed in this paper is an interactive labeling method which combines the characteristics of the image itself with manual labeling operations so that better segmentation results can be obtained.

## 5. Conclusions

Although the current deep learning method represented by Mask R-CNN has achieved high-pixel-level segmentation accuracy, it is based on training via inputting masks. At present, these masks are made manually. When the object boundary is very complex and the dataset is especially large, this consumes time and energy. Therefore, we proposed a mask-making method based on GRABCUT which can quickly obtain masks for object detection.

Experiments on the BigBIRD (Big Berkeley Instance Recognition Dataset) verified the effectiveness of our proposed method, which achieved a mAP index of over 95% for segmentation. While maintaining the positioning and segmentation performance of Mask R-CNN, this method ensures that the required mask can be obtained simply and efficiently. We also extended our experiments to the COCO dataset and electronic component solder joint defect detection to further prove the effectiveness of our proposed method.

The proposed method can also be applied to other object recognition tasks and can be easily generalized to other fields that require image annotation. Although the efficiency of our proposed method is improved compared with that of the manual annotation method, it still requires some labeling and image conversion operations; thus, we will focus on these issues in the future to achieve real automatic mask acquisition.

## Figures and Tables

**Table 1:** Results of recognition accuracy with different datasets.

Dataset	mAPbbox
ikea_table_leg_blue;	99.99%
ikea_table_red_cup	98.26%
3 m_high_tack_spray_adhesive	98.16%
Person objects in COCO dataset	99.8%
Pizza objects in COCO dataset	99.3%

**Table 2:** Comparison of recognition accuracy and target detection results of different methods.

Method	Accuracy	mAP
Principal component analysis	96.43%	--
Support vector machine	96.43%	--
Ensemble learning	89.29%	--
Mask R-CNN with manual label	100%	97.4%
Our proposed method	100%	98.5%
